# Parenchymal Neuro-Behçet disease with heterogeneous clinical presentations: a comparative case report

**DOI:** 10.3389/fimmu.2026.1965989

**Published:** 2026-10-09

**Authors:** Alaa Naiem Zaro, Yumna Alatrash, Raya N. Amro, Mohammed Y. Nairoukh, Nidal Itmaiza, Ikram Qaysia

**Affiliations:** 1 Faculty of Medicine, Al-Quds University, Jerusalem, Palestine; 2 Alia Hospital, Hebron, Palestine; 3 Department of Radiology, Alia Hospital, Hebron, Palestine; 4 Department of Internal Medicine, Alia Hospital, Hebron, Palestine

**Keywords:** Behçet disease, brainstem syndrome, comparative case report, immunosuppressive agents, magnetic resonance imaging, Neuro-Behçet disease

## Abstract

**Background:**

Neuro-Behçet disease (NBD) is a rare but potentially disabling manifestation of Behçet disease (BD). Its heterogeneous neurological presentation can mimic central nervous system infection, cerebrovascular disease, inflammatory demyelination or intracranial tumors, making early diagnosis difficult. Importantly, there is still limited published data on NBD from Palestine regarding presentation, diagnosis, imaging and management.

**Case presentation:**

We describe two patients with a long history of recurrent oral and genital ulceration who were diagnosed with parenchymal Neuro-Behçet disease (P-NBD). The first patient was a 26-year-old man with a two-week progressive headache, fever, vomiting, and blurred vision, which resembled meningitis, in whom examination showed central facial weakness, gait imbalance and pathological laughter. The second patient was a 41-year-old woman with a two-year history of gait imbalance and dysarthria who presented with three days of dizziness, horizontal nystagmus, central facial weakness, right lower-limb weakness, and a positive bilateral extensor plantar response. Laboratory testing and brain magnetic resonance imaging (MRI) were performed to exclude other differential diagnoses. Both received intravenous methylprednisolone followed by an immunosuppressive agent.

**Results:**

The first patient had clinical and radiological improvement after corticosteroids and initiation of induction cyclophosphamide, with clinical improvement on follow-up. The second patient improved significantly on imaging after infliximab maintenance therapy. She also improved clinically, but chronic symptoms of gait imbalance and dysarthria were still present.

**Conclusion:**

These two cases illustrate the variable presentation of P-NBD, including a meningitis-like febrile syndrome with pathological laughter and an acute-on-chronic brainstem syndrome. In patients with recurrent oro-genital ulceration and unexplained brainstem or diencephalic neurological findings, NBD should be considered after careful exclusion of other disorders. Early diagnosis and immunosuppressive treatment can lead to substantial clinical and radiological improvement.

## 1 Introduction

Behçet disease (BD) is a chronic, multisystemic, variable-vessel vasculitis of unknown etiology characterized by a relapsing course. Its reported prevalence varies markedly with geography: the pooled global estimate is 10.3 per 100,000 population (95% CI 6.1–17.7) (1), and the disease is concentrated along the historical Silk Road (1, 2). A recent systematic review and meta-analysis of the Middle East and North Africa reported a pooled regional prevalence of approximately 300 per 100,000 population, among the highest worldwide (3). The classical manifestations include recurrent oral and genital ulcers, skin hypersensitivity and lesions, and ocular inflammation, with variable involvement of other systems, including the joints, vasculature, the gastrointestinal system, and even the nervous system. Diagnosis of BD is primarily clinical, as there is no pathognomonic laboratory test or definitive diagnostic test. Nowadays, much evidence suggests that environmental and infectious factors in genetically susceptible individuals trigger BD (1). Due to its diverse manifestations and wide geographical distribution, it has a global health impact (1, 2).

Neurological involvement (i.e., Neuro-Behçet disease [NBD]) is one of the most serious manifestations of BD and a major cause of long-term disability. It has been reported in approximately 5–10% of patients in large series (4), with higher pooled estimates in high-prevalence regions, for example, 18.54% (95% CI 13.93–24.25%) across 4,262 North African patients with BD (5), and 10.3% in a Lebanese tertiary-centre cohort (6).

Central nervous system involvement in NBD is divided into a parenchymal form (P-NBD), which affects parenchymal structures (e.g., the brainstem, diencephalon, and cerebral hemispheres), and a non-parenchymal form (NP-NBD), which encompasses cerebral venous sinus thrombosis, aseptic meningitis, and intracranial hypertension. Peripheral nervous system involvement is rare (7). Early recognition of NBD is required to limit long-term complications and death (8).

The meningeal irritation in NBD is a particular diagnostic challenge. Its manifestations and cerebrospinal fluid (CSF) features overlap with infectious meningitis and occasionally with neoplastic and demyelinating disease. So, a diagnosis of exclusion in these situations is often required and is established by repeated sterile CSF analysis, a negative infectious workup, evidence of NBD on imaging, and the systemic features of BD (9).

Although several case reports have been published in multiple countries, there is a shortage of published NBD cases in the Palestinian territories in particular; no dedicated NBD series has been identified. To address this gap, the article presents a comparative case report of two cases of NBD diagnosed at Alia Hospital in Hebron, Palestine. This report highlights two contrasting presentations of parenchymal Neuro-Behçet disease: a meningitis-like febrile syndrome with pathological laughter in Case 1 and an acute neurological deterioration superimposed on a chronic progressive brainstem syndrome in Case 2. Both cases demonstrated characteristic brainstem–diencephalic MRI involvement, including mesodiencephalic extension, illustrating how markedly different clinical presentations may converge on a similar radiological phenotype.

## 2 Case presentation

### 2.1 Case 1 presentation

A 26-year-old male, a 10-pack-year smoker from the West Bank with an otherwise unremarkable medical history and no history of alcohol consumption, was admitted to the internal medicine department with a headache of two weeks’ duration. He had been in his usual state of health until two weeks before presentation, when he developed a progressively worsening headache. It increased in intensity over time, was not relieved by simple analgesics, and reached approximately 6/10 in severity. It was associated with nausea, two episodes of vomiting, blurred vision, and documented fever up to 39 °C. There was no photophobia or neck stiffness, seizure activity, rash, diarrhea, weight loss, night sweats, recent travel, or known tuberculosis contact.

Six months before the presentation, he had experienced recurrent episodes of headache associated with blurred vision and ocular dryness. He also reported a two-year history of recurrent oral (more than 3 times per year) and genital ulcers, together with polyarthralgia. A pathergy test was positive in an external clinic. Against this background, BD was clinically suspected.

On admission, the temperature was 38.5 °C. He was conscious, alert, and oriented but exhibited episodes of inappropriate, involuntary laughter (pathological laughter). The Glasgow Coma Scale score was 15/15 (E4V5M6). Cardiovascular, respiratory, and abdominal examinations were unremarkable: the abdomen was soft and non-tender, and breath sounds were normal bilaterally with good air entry. No skin rash and no active oral or genital ulcers were seen at the time of examination.

Neurological examination showed normal muscle power (5/5) in all four limbs with intact sensation. A left central (upper motor neuron [UMN]) facial palsy was documented on the neurology assessment. There was no neck stiffness and no signs of meningeal irritation. Due to brainstem localization, gait imbalance was also noted during the illness. The patient had a modified Rankin Scale (mRS) score of 3, reflecting moderate neurological disability due to UMN facial palsy, brainstem dysfunction, and gait imbalance.

#### 2.1.1 Laboratory findings on admission

High-sensitivity C-reactive protein was normal (2.2 mg/L, falling to <1.0 mg/L; reference <5 mg/L) and the erythrocyte sedimentation rate was elevated (20 mm/h; reference 0–15 mm/h). Hepatitis B surface antigen, hepatitis C antibody, and HIV serology were all non-reactive. The admission complete blood count showed leukocytosis (white cell count 12.2 ×10^9^/L, rising to 24.3 ×10^9^/L during steroid pulse therapy) with neutrophil predominance; hemoglobin, platelets, renal function, and electrolytes were within or close to normal limits. Liver transaminases were mildly elevated (peak ALT 45 U/L). Coagulation studies (INR 1.05, APTT 32.6 s) and D-dimer (270 ng/mL) were normal. Complement C3 (127) and C4 (33.3) were normal, and rheumatoid factor was negative; Brucella (Rose Bengal) was negative. Glycated hemoglobin was 5.2%, thyroid-stimulating hormone (2.84) and vitamin B12 (354 pg/mL) were normal, and 25-hydroxyvitamin D was low (18.4 ng/mL). Per the hospital’s infectious workup protocol, two sets of blood cultures were obtained from separate venipuncture sites; both showed no growth.

Two lumbar punctures were performed during the course. Both yielded clear cerebrospinal fluid with normal glucose and protein, no organisms on Gram stain, and no growth on culture (**Table 1**), arguing against pyogenic bacterial meningitis. The first sample showed a mild pleocytosis (10 cells/µL, 70% neutrophils), consistent with the aseptic inflammatory picture of NBD; a second lumbar puncture, performed during the third admission on 10 June 2026 before cyclophosphamide initiation, showed no white blood cells and 20 RBC/µL, which can be traumatic. Opening pressure was not measured at either tap.

**Table 1:** Cerebrospinal fluid (CSF) analysis, case 1.

CSF parameter	LP1 (17/05/2026)	LP2 (10/06/2026)	Reference
Appearance	Clear, colourless	Clear	—
White cell count (/µL)	10	0	0–5
Red cell count (/µL)	—	20	—
Neutrophils	70%	0	—
Lymphocytes	30%	0	—
Protein (mg/dL)	27.4	28.4	15–45
Glucose (mg/dL)	55	58	40–70
Gram stain	Negative	Negative	—
Culture	No growth	No growth	—

LP1, first lumbar puncture (LP), performed during the first admission; LP2, second lumbar puncture, performed during the third admission before cyclophosphamide initiation.

#### 2.1.2 Imaging

A CT scan showed a hypodense lesion in the parietal region and midbrain, so a brain MRI (17/05/2026, with and without contrast) was done and demonstrated T2/fluid-attenuated inversion recovery (FLAIR) hyperintense signal involving the right basal ganglia with patchy contrast enhancement in the right basal ganglia and right thalamus (**Figures 1A, B**). Also, in the internal capsules (more pronounced on the right), the midbrain (**Figure 1C**), and the right side of the pons. An abnormal T1-weighted image revealed mild patchy enhancement on the coronal section (**Figure 1D**). No diffusion restriction was identified on diffusion-weighted imaging (DWI), which argues against acute infarction as the cause of the deficit. The reporting radiologist considered the appearances highly suggestive of NBD.

**Figure 1:**
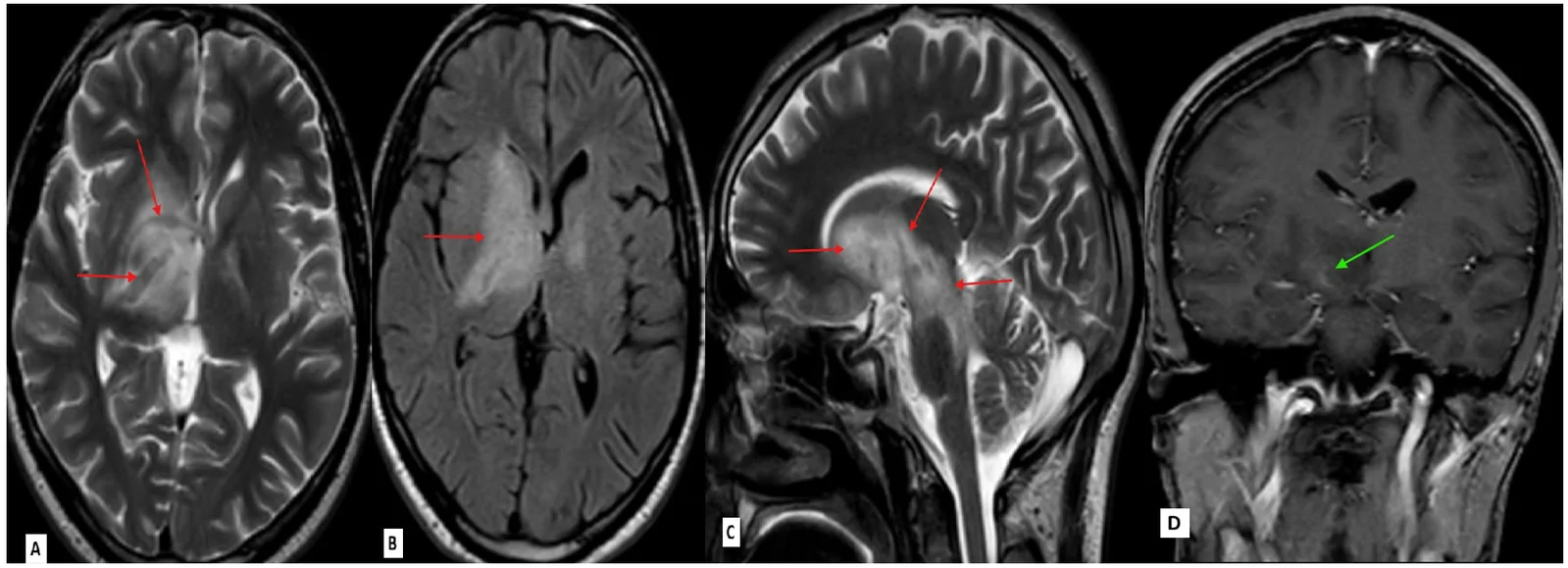
Brain MRI demonstrating parenchymal Neuro-Behçet disease. **(A, B)** Axial T2-weighted/FLAIR images show a hyperintense lesion involving the right thalamus and basal ganglia. **(C)** Sagittal T2-weighted image demonstrates longitudinal extension of the lesion from the basal ganglia through the mesodiencephalic junction into the midbrain (red arrows). **(D)** Coronal contrast-enhanced T1-weighted image reveals mild patchy enhancement (green arrow).

To exclude an occult malignant or infective focus before cytotoxic therapy, contrast-enhanced CT of the neck, chest, abdomen, and pelvis was performed (03/06/2026); it was unremarkable, with no mass lesion, lymphadenopathy, effusion, or metastatic disease. A repeat non-contrast brain CT before the second lumbar puncture showed no acute change, intracranial hemorrhage, mass lesion, or significant mass effect.

#### 2.1.3 Management and course

The patient received pulsed intravenous methylprednisolone (Solu-Medrol) 1 g daily for five days. Because escalation to cytotoxic therapy (cyclophosphamide) was planned but the patient still had fever, cyclophosphamide was deliberately withheld until repeat lumbar puncture and imaging showed no active bacterial infection or another systemic infectious focus. This staged approach spanned three admissions: the first for diagnosis and steroid pulse therapy (17–21 May); a second (25 May–5 June) during which the rheumatology service advised confirming a sterile CSF and a clear whole-body CT before immunosuppression, during which the leukocytosis was attributed to steroid therapy rather than active pyogenic infection; and a third (8–13 June) at which, following a sterile repeat lumbar puncture and reassuring imaging, cyclophosphamide was initiated (counted as day 1).

Cyclophosphamide was then initiated as an intravenous infusion of 1000 mg every four weeks, planned for six months as induction therapy, along with tapering oral corticosteroids. Each infusion was given with adequate intravenous hydration before and after administration, antiemetic prophylaxis, and mesna to prevent hemorrhagic cystitis. The patient improved clinically, and a follow-up imaging study performed before the subsequent cyclophosphamide dose demonstrated improvement of the previously described lesions, with no evidence of active inflammatory disease. He was discharged with a planned follow-up in the rheumatology, neurology, and ophthalmology clinics.

On follow-up in outpatient clinics, his functional disability scores significantly improved to an mRS of 1. The patient still had leukocytosis (white cell count 13.7 × 10^9^/L) with neutrophilic predominance. But there was no fever or other signs of infection, and the patient improved clinically. Ophthalmologically, no ophthalmic signs of BD were found. The patient denied experiencing any side effects from the medication.

### 2.2 Case 2 presentation

A 41-year-old woman, a known case of type 2 diabetes mellitus (T2DM) controlled by metformin without any microvascular complications contributing to her diabetes, had been in her usual state of health, though already limited by an unsteady gait and dysarthria for 2 years, until three days before admission. She was admitted to the emergency department with a three-day history of dizziness and fatigue. It was associated with deviation of the mouth to the left, numbness over the entire left side of the body, gait imbalance, and right-sided weakness. There was no loss of consciousness, blurred vision, nausea, vomiting, involuntary movements, sphincter dysfunction, fever, or palpitations.

Neurological examination showed the patient was conscious and alert with a Glasgow Coma Scale score of 15/15. Both pupils were reactive to light, horizontal nystagmus was noted, and speech was dysarthric. Head and neck examination showed a normal range of motion without deformity. Facial examination revealed deviation of the mouth to the left with preserved forehead wrinkling, indicating an upper-motor-neuron (central) facial pattern. On motor examination, bulk, tone, and deep-tendon reflexes were normal in all four limbs; power was full (5/5) in both upper limbs and in the left lower limb, with mild weakness (4/5) in the right lower limb. Sensation was preserved in all limbs. Plantar responses were extensor (positive Babinski) bilaterally. The gait was unsteady with incoordination, while the Romberg test was negative. Lower-limb examination showed no oedema and no clinical signs of deep vein thrombosis.

The patient’s functional impairment corresponded to a mRS score of 3, reflecting moderate multi-system neurological disability secondary to pyramidal, brainstem, and cerebellar involvement.

#### 2.2.1 Laboratory findings on admission

Laboratory investigations showed a markedly elevated erythrocyte sedimentation rate (66 mm/h) and a raised C-reactive protein level (10.1 mg/L). HIV and hepatitis C serology were negative, and the thyroid-stimulating hormone was normal. Transthoracic echocardiography (24/05/2026) showed normal left- and right-ventricular size and function (ejection fraction ~65%), minimal mitral-valve prolapse with trace regurgitation, and a mild pericardial effusion of no hemodynamic significance; a non-contrast chest CT confirmed no lung mass, mediastinal lymphadenopathy, or pleural effusion, with the same mild pericardial effusion.

#### 2.2.2 Imaging

A non-contrast CT of the brain showed no intracranial hemorrhage or midline shift, but findings suggestive of right mastoiditis (ENT referral was advised), with minimal fluid in the right mastoid air cells.

The subsequent MRI (24/05/2026) showed T2/FLAIR hyperintensity in the left basal ganglia, left thalamus, and left midbrain, with patchy enhancement (**Figures 2A, B**). On the sagittal view, there was longitudinal extension of the lesion from the basal ganglia through the midbrain into the upper pons (**Figure 2C**). Additionally, the coronal view demonstrated abnormal signal extending through the mesodiencephalic junction, consistent with the radiological cascade (waterfall) sign (**Figures 2D, E**). A T1-weighted MRI image was also obtained and revealed moderate patchy enhancement on the coronal view (**Figure 2F**). Although ischemic stroke was initially considered in the differential diagnosis because of the acute focal signs and the inflammatory changes on imaging, no diffusion-restricted infarct was demonstrated on DWI. The appearances were considered highly suggestive of NBD, with infiltrative tumor thought less likely, and follow-up MRI in two months was advised.

**Figure 2:**
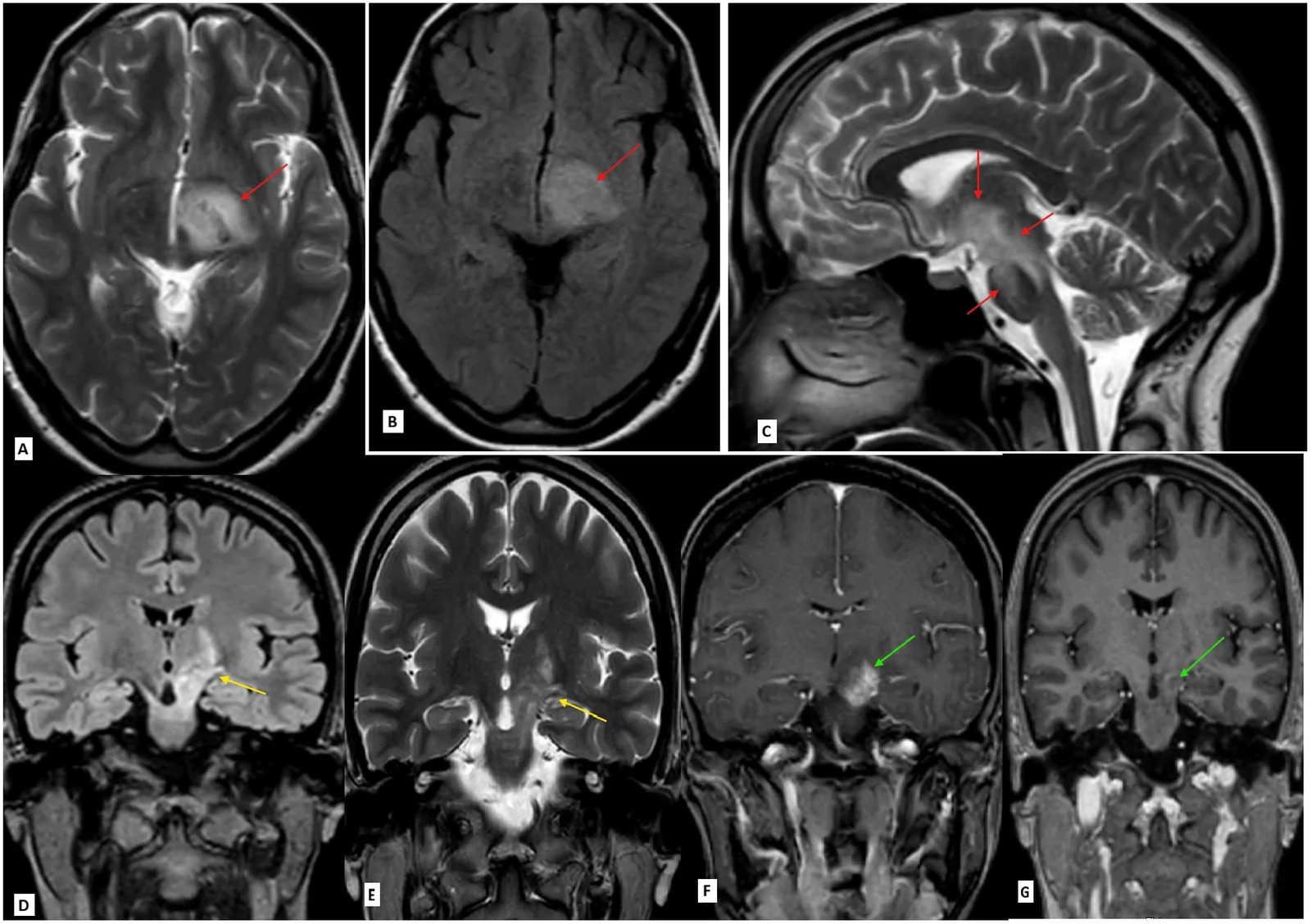
Brain MRI demonstrating characteristic parenchymal Neuro-Behçet disease. **(A, B)** Axial T2-weighted/FLAIR images show a hyperintense lesion involving the left basal ganglia. **(C)** Sagittal T2-weighted image demonstrates longitudinal extension of the lesion from the basal ganglia through the midbrain into the upper pons (red arrows). **(D, E)** Coronal FLAIR/T2-weighted images demonstrate abnormal signal extending through the mesodiencephalic junction, consistent with cascade or “waterfall” sign (yellow arrow). **(F)** Coronal contrast-enhanced T1-weighted image reveals moderate patchy enhancement (green arrow). **(G)** Follow-up coronal post-contrast T1-weighted image obtained 2 months after treatment demonstrates minimal residual enhancement, consistent with significant radiological improvement and reduced parenchymal inflammatory activity.

#### 2.2.3 Further clinical history

The history of BD was obtained only after the MRI findings raised the possibility of NBD, and a focused history was obtained. The patient reported a five-year history of recurrent oral and genital ulcers and skin hypersensitivity, with papulopustular lesions according to previous dermatological reports, two years of gait imbalance, and a 10-day history of right-sided facial palsy with tongue deviation, right-sided weakness, and incoordination, the neurological illness having evolved subacutely over days on a background of chronic mucocutaneous disease spanning years.

#### 2.2.4 Management and course

Based on the clinical history, neurological findings, MRI appearances, and history of recurrent orogenital ulcers, a diagnosis of NBD was made. The patient received intravenous methylprednisolone (Solu-Medrol) 1 g daily as pulse therapy for five days. Clinical improvement was evident by the third day and continued to the fifth. The first complaint that improved with steroids was fatigue. She was discharged on a tapering course of oral prednisolone (20 mg three times daily for seven days, then reducing by 10 mg every seven days until discontinuation), with calcium and vitamin D supplementation for steroid-related bone protection, and a rheumatology consultation was arranged for ongoing immunosuppressive management.

On rheumatology consultation, the patient was advised to start infliximab at 5 mg/kg at weeks 0, 2, and 6, then every 6 weeks, in addition to prednisone tablets. Tuberculosis and hepatitis B screening were performed before starting anti-TNF-α therapy, and an ENT specialist excluded mastoiditis as a source before immunosuppression. The patient started to improve clinically, and a follow-up brain post-contrast T1-weighted MRI after 2 months of treatment demonstrated significant resolution of the previously described lesions (**Figure 2G**). She also initiated physiotherapy for walking and speech rehabilitation. At the two-month follow-up, her disability score improved from an mRS of 3 on admission to an mRS score of 2. The patient denied experiencing any side effects from the medication.

**Table 2** summarizes the admissions and clinical timeline for both cases.

**Table 2:** Diagnostic criteria, admissions and clinical timeline for both cases.

Case	BD/NBD diagnostic criteria fulfilled	Admission	Dates	Reason/presentation	Working diagnosis	Management
1	BD: recurrent oral ulcers, recurrent genital ulcers, positive pathergy. NBD: compatible parenchymal neurological syndrome, characteristic MRI, supportive CSF findings.	1	17–21 May 2026	Headache 2 weeks, fever, vomiting, blurred vision, inappropriate (pathological) laughter	Neuro-Behçet disease	IV methylprednisolone 1 g/day ×5; discharged for rheumatology review
		2	25 May–5 Jun 2026	Readmitted for planned cytotoxic therapy; rheumatology advised excluding infection first	Bacterial vs aseptic meningitis; leukocytosis attributed to steroids	No evidence of pyogenic meningitis and systemic infectious focus (sterile repeat CSF, clear whole-body CT); discharged on home meds
		3	8–13 Jun 2026	Admitted to initiate cyclophosphamide	Neuro-Behçet disease	Cyclophosphamide started (Day 1); follow-up imaging showed improvement
2	BD: recurrent oral ulcers, recurrent genital ulcers, papulopustular skin lesions. NBD: compatible brainstem/cerebellar/pyramidal syndrome, characteristic MRI.	1	23–28 May 2026 (imaging & echo 24/05/2026)	3-day dizziness with leftward mouth deviation, left hemibody numbness, right-sided weakness, gait imbalance; background 5-yr orogenital ulcers, 2-yr gait imbalance/dysarthria, 10-day right facial palsy	Neuro-Behçet disease (infiltrative tumor and ischemic stroke considered less likely)	IV methylprednisolone 1 g/day ×5 (improvement by day 3); discharged on tapering oral prednisolone + calcium/vitamin D; infliximab as a maintenance therapy

## 3 Literature review and discussion

We have reported two cases who received a P-NBD diagnosis from the West Bank according to exclusion-based workup and imaging findings after a subacute brainstem–diencephalic syndrome developed on a background of long-standing recurrent orogenital ulceration.

### 3.1 Definition, epidemiology and regional burden

BD is a chronic, relapsing, multisystemic, variable-vessel vasculitis that affects both arteries and veins, defined clinically by the presence of oral and genital ulcers, ocular inflammation and skin lesions. Its geographical distribution along the historical Silk Road gives it one of the more distinctive epidemiologies in medicine (1, 2).

Although multiple genetic and environmental factors have been implicated in the disease’s pathogenesis, the exact cause remains unknown. Multiple genes, most of which are involved in the inflammatory response, have been identified. HLA-B*51 (serologically HLA-B51, within the broader HLA-B5 antigen group) has the strongest genetic association (10).

Neurological involvement is reported at widely varying frequencies across series. The prevalence of BD is highest in the Middle East and North Africa, where pooled regional estimates are among the highest reported worldwide (3), making an NBD diagnosis more likely in patients from these areas than figures derived from Europe or North America would suggest. Because BD is highly prevalent in the Middle East, the probability of NBD among patients in the West Bank is higher than in other regions. Nevertheless, regional evidence remains sparse (11).

### 3.2 Clinical spectrum of Neuro-Behçet disease

A recent systematic review of NBD reinforced that early clinical and imaging features are heterogeneous and that the evidence base is dominated by case reports and single-center series (12). It typically emerges years after the systemic onset of BD, affecting males more than females but with higher severity in females (13). The parenchymal type (P-NBD) can be subdivided into two distinct groups based on presentation. Acute P-NBD (A-P-NBD) presents with fever, hemiparesis, and an elevated CSF cell count. In contrast, chronic progressive P-NBD (CP-P-NBD) is characterized by ataxia, dementia, incontinence, and brainstem atrophy. However, it is sometimes difficult to categorize every case as either acute or chronic progressive. A proportion of A-P-NBD can turn into secondary chronic progressive with time.

On the other hand, the non-parenchymal type (NP-NBD) manifests as cerebral venous thrombosis, leading to severe headache, papilledema, and features of elevated intracranial pressure (7, 8). The clinical course can range from a single self-limited attack to many recurrent relapses with progressive and long-term neurological disability.

### 3.3 Diagnostic framework of Neuro-Behçet disease

Since there is no pathognomonic test for BD, the diagnosis relies on the 1990 International Study Group (ISG) criteria (14). It defines BD as recurrent oral ulcers (at least 3 times/year) with at least two of the following: recurrent genital ulcers, ocular lesions, skin lesions, or a positive pathergy test. Accordingly, the diagnosis of NBD relies on recognizing a compatible neurological syndrome in a patient who already meets the criteria for BD. The most widely used diagnostic framework for NBD, categorizing it into P-NBD and NP-NBD, is the International Consensus Recommendations (ICR) (8).

ICR classified NBD as either a definite or a probable diagnosis. A definite diagnosis requires the fulfilment of the ISG criteria for BD, the presence of a recognized neurological syndrome, and compatible MRI and/or CSF abnormalities, with exclusion of an alternative diagnosis. Probable NBD includes patients with a characteristic neurological syndrome but do not fulfil the ISG criteria or patients with full ISG criteria but present with atypical neurological manifestations (8). Both of our patients fulfilled the ISG criteria and have a definite NBD diagnosis according to ICR.

### 3.4 Imaging in Neuro-Behçet disease

MRI is the imaging modality of choice for NBD and is more sensitive than CT. Its yield depends on the clinical phenotype: in a series of 104 patients with BD and neurological involvement, parenchymal lesions most often involved the brainstem (84.8%), the mesodiencephalic junction (77.9%) and the basal ganglia (68.6%) (11), whereas cranial MRI was normal in almost half of the examinations of patients with primary progressive or clinically silent CNS disease (15). The imaging findings will be grouped into parenchymal disease and non-parenchymal disease.

Parenchymal disease. Acute and subacute lesions appear as focal areas of high signal on T2-MRI and FLAIR images, often with variable contrast enhancement. The most common site of lesions is the brainstem (mainly the midbrain and pons), which can extend into the diencephalon (thalamus, basal ganglia) across the mesodiencephalic junction; a mesodiencephalic junction lesion with extension along long tracts was the commonest MRI finding (46%) in the original series and is highly suggestive of P-NBD (4). This pattern of extension from the thalamus to the midbrain has been termed the “cascade” or “waterfall” sign (16), and is clearly illustrated in this case report.

Additional sites include the cerebral white matter, the internal capsule and, less often, the cerebral cortex (4, 11). Repeated attacks can lead to brainstem atrophy, which was documented in 15 patients examined more than one year after an initial parenchymal CNS episode, predominantly in those with a secondary progressive course (15); chronic NBD may also show brain or brainstem atrophy and “black holes” on MRI, a hallmark of CP-P-NBD (16).

Non-parenchymal disease. MR venography (MRV) or CT venography demonstrating cerebral venous sinus thrombosis is the hallmark of the non-parenchymal form (16). Among the 18 patients with cerebral venous thrombosis in the Moroccan series, involvement was of the superior sagittal sinus alone in 44.4%, the lateral sinus alone in 22.2%, and of both sinuses concomitantly in 16.6% (11).

In this paper, the imaging findings of localization to the brainstem and mesodiencephalic junction, with additional involvement of the basal ganglia, thalamus, and internal capsule, reproduce the above-mentioned pattern of P-NBD on T2/FLAIR imaging (4).

### 3.5 Treatment in Neuro-Behçet disease

No large randomised clinical trials exist; management is mainly guided by the 2025 EULAR recommendations, supplemented by observational data and expert opinion (17, 18).

Acute Parenchymal attack. High-dose glucocorticoids, as follows: IV methylprednisolone, 1g/day for 5–7 days, followed by an oral prednisolone taper over 2–3 months (17).

Maintenance Therapy. Because the disease relapses, glucocorticoids alone are insufficient, and an additional agent should be considered. Tumor necrosis factor alpha (TNF-α) inhibitors (mainly infliximab) are commonly used as first-line therapy, nowadays, supported by the systematic literature review underpinning the EULAR recommendations (17, 18). Observational evidence also supports the use of anti-TNF therapy in severe or refractory NBD; Desbois et al. reported favorable clinical outcomes with anti-TNF agents in this setting (19). Cyclosporine has a neurotoxic effect and an association with CNS manifestations in BD; hence, it should be avoided (7, 17). Azathioprine remains a conventional immunosuppressive option but is no longer recommended as a first-line agent and should not necessarily be used before other agents (17).

In patients with cerebral venous sinus thrombosis, glucocorticoids can control the underlying inflammation. Anticoagulation may be added for venous thrombosis after careful screening for coexisting aneurysms, a distinctive caution in BD (17).

Management in both cases followed the glucocorticoid-based strategy recommended for acute parenchymal attacks (17, 18). Because relapse is expected, immunosuppression was escalated with cyclophosphamide in Case 1 and with the TNF-α inhibitor infliximab in Case 2 (17). Neither of the patients received azathioprine. The 2025 EULAR recommendations support early use of TNF-α inhibitors in P-NBD (17, 18). Although the 2018 EULAR recommendations listed cyclophosphamide among conventional immunosuppressive options for severe disease (20), its role has since narrowed. However, the patient received it in Case 1 because biological therapies were limited in availability in the country.

### 3.6 Two-case comparison

The educational value of these cases lies primarily in their contrasting clinical presentations rather than in comparison of treatment efficacy. Case 1 illustrates a febrile, meningitis-like presentation accompanied by pathological laughter, whereas Case 2 demonstrates an acute neurological deterioration superimposed on a two-year history of gait imbalance and dysarthria. These two patients also differ in age, disease duration, comorbidity and treatment, so the difference in severity cannot be attributed to sex; the reported tendency of the parenchymal form to be more severe in women, although more frequent in men, is noted here only as a hypothesis-generating observation (7, 12).

The inflammatory profile and imaging also diverged. Imaging in both localized to the brainstem and mesodiencephalic junction with basal-ganglia and thalamic extension, but the lesions were right-predominant in Case 1 and left-predominant in Case 2, the latter additionally showing acute inflammatory changes, with acute ischemic infarction considered unlikely because no diffusion restriction was demonstrated on DWI (4). The leading alternative diagnosis differed accordingly: infective meningitis in Case 1 and infiltrative tumor or ischemic stroke in Case 2, yet both required the same exclusion-based, multidisciplinary workup before immunosuppression. The differing immunosuppressive regimens reflect individualized clinical management and should not be interpreted as comparative evidence of treatment effectiveness. Together, these cases show how a single parenchymal phenotype can present, and be managed, along a spectrum determined by acuity, inflammatory burden, and lesion laterality.

## 4 Patient perspective

The first patient found the diagnosis unexpected; his prior disease had been confined to recurrent oral ulcers and arthralgia, and a neurological complication had seemed remote. The acute illness disrupted his work and daily activities, but after corticosteroid and cyclophosphamide therapy he recovered well and returned to full employment and independent living. He described himself as resilient, emphasizing the strong support he received from his family throughout his illness.

The second patient reported a heavier physical and emotional burden, reflecting the chronic, progressive course of her mobility and speech impairment. She credited the sustained support of her husband, together with her own patience and determination, for her adherence to treatment and rehabilitation, including physiotherapy and speech therapy. She also highlighted the difficulty of obtaining infliximab in Palestine, where recurrent interruptions in drug availability delayed scheduled infusions and added anxiety to her treatment course.

## 5 Conclusion

We present two cases illustrating the diagnostic difficulty and clinical heterogeneity of P-NBD. They both developed brainstem-diencephalic neurological syndromes on a background of recurrent oral and genital ulceration, but their initial presentations were quite different. The diagnosis was supported by the characteristic radiological involvement pattern on MRI and the exclusion of other causes. Both patients improved following high-dose intravenous corticosteroid therapy. NBD should therefore continue to be considered an important differential diagnosis in patients from regions where BD is endemic presenting with unexplained brainstem or diencephalic syndromes, especially if there is recurrent oro-genital ulceration. This study had several limitations. First, the limited follow-up period precludes assessment of long-term complications that may occur several years after diagnosis. Second, we did not perform genetic testing for the HLA-B*51 allele, although it is not required for diagnosis. Third, we did not document dedicated testing for neurotropic viruses, tuberculosis, or fungal infections, limiting comprehensive exclusion of infectious causes; we also used cyclophosphamide instead of infliximab in Case 1. Finally, for future papers, we recommend doing a larger case series with a prospective cohort and long-term follow-up, which can effectively increase the chance of timely diagnosis, safe exclusion of competing conditions, and selection of appropriate immunosuppressive therapy.

## Data Availability

The original contributions presented in the study are included in the article/supplementary material. Further inquiries can be directed to the corresponding authors.
